# Prediction of hemorrhagic transformation after thrombolysis based on machine learning models combined with platelet distribution width-to-count ratio

**DOI:** 10.3389/fneur.2025.1466543

**Published:** 2025-10-02

**Authors:** Xiaosheng Li, Chunyan Lei, Hongyun Xu, Churan Yuan, Yuzhen Zhou, Wen Jiang

**Affiliations:** ^1^Department of Neurology I, First Affiliated Hospital of Kunming Medical University, Kunming, China; ^2^Department of Neurology, The 920th Hospital of Joint Logistics Support Force, Kunming, China; ^3^Yunnan Clinical Center for Neurological and Cardiovascular Diseases, Kunming, China; ^4^Department of Endocrinology, Xuanwei Hospital Affiliated to Yunnan University of Chinese Medicine, Xuanwei, China; ^5^Department of Rehabilitation Medicine, The Affiliated Hospital of Yunnan University, Kunming, China

**Keywords:** hemorrhagic transformation, intravenous thrombolytic therapy, stroke, platelet distribution width-to-count ratio, machine learning

## Abstract

**Background:**

Hemorrhagic transformation (HT) is a common and potentially serious complication following intravenous thrombolysis (IVT) in patients with acute ischemic stroke (AIS). Despite its high incidence, there remains a lack of simple and effective tools for predicting HT risk.

**Objective:**

This study aimed to develop an interpretable machine learning (ML) model incorporating the platelet distribution width to platelet count ratio (PPR) to predict HT occurrence in AIS patients after IVT.

**Methods:**

We included AIS patients who underwent IVT at the First Affiliated Hospital of Kunming Medical University between July 2019 and April 2024. Four ML models—logistic regression (LR), random forest (RF), support vector machine (SVM), and extreme gradient boosting (Xgboost)—were constructed using 5-fold cross-validation, with HT after IVT as the outcome. Feature selection was performed using least absolute shrinkage and selection operator (LASSO) regression. Model performance was evaluated based on the area under the receiver operating characteristic curve (AUC), accuracy, sensitivity, specificity, and balanced F-score. The best-performing model was selected for interpretability analysis, and feature importance was assessed.

**Results:**

LASSO regression identified six predictive features with non-zero coefficients: age, diabetes, malignancy, onset-to-treatment time (OTT), baseline National Institutes of Health Stroke Scale (NIHSS) score, and PPR. Among the models, LR demonstrated the highest predictive performance, achieving an optimal AUC of 0.919, along with average accuracy, sensitivity, and specificity of 0.825, 0.830, and 0.832, respectively. Feature importance in the LR model ranked as follows: baseline NIHSS score, diabetes, PPR, malignancy, age, and OTT.

**Conclusion:**

The LR-based model incorporating PPR effectively predicts HT risk in AIS patients after IVT, providing clinicians with a rapid and accurate tool to assess thrombolytic hemorrhage risk and support treatment decision-making.

## 1 Introduction

Alteplase intravenous thrombolytic therapy (IVT) is effective in treating acute ischemic stroke (AIS) (1). Hemorrhagic transformation (HT) is a frequently occurring potentially adverse complication of intravenous thrombolysis in AIS patients, and the occurrence of HT in AIS patients following thrombolysis is significantly higher than that in AIS patients without thrombolysis (2). HT is further grouped into hemorrhagic infarction (HI) and parenchymal hemorrhage (PH), where PH, as a progressive manifestation of HI, usually indicates poor prognosis (3–5). Therefore, early identification of potential HT during thrombolysis and asymptomatic HI patients after thrombolysis is crucial.

Currently, most of the studies use multiple linear regression (MLR) to identify risk factors (6, 7) for HT following thrombolysis, including white blood cells (8), coagulation function (9), bilirubin (10), and uric acid (11). However, MLR is restrained by linear hypotheses between predicted variables and results, and the sensitivity to outliers may have adverse effects on predictive performance (12). Machine learning (ML), as an emerging discipline in the medical field, leverages computer science and statistical techniques to address healthcare challenges (13), making up for the shortcomings mentioned above and thus being widely applied. Due to the varying performance of different ML algorithms in different application scenarios, it is necessary to select appropriate algorithms to optimize model performance and accuracy before constructing a risk model for predicting HT after thrombolysis.

Platelet is the main component of blood and plays a crucial role in the onset and progression of AIS by maintaining the integrity of vascular endothelial cells, coagulation, and other pathophysiological functions (14, 15). Platelet count changes in hemorrhagic diseases are typically more rapid and pronounced than fibrinolytic markers. Thrombocytopenia or dysfunction is often an early sign of bleeding, while fibrinolytic indicators require some time to accumulate before showing any changes, which may not reflect risks in the early stages of HT. Additionally, fibrinolytic markers can be influenced by more complex factors, such as liver dysfunction and inflammatory responses (16), which can limit their clinical utility. These markers primarily reflect the degree of fibrinolytic activity, and although fibrin degradation products and D-dimer have some predictive value for HT after IVT (17, 18), their accuracy and timeliness are inferior to platelet count. Studies has demonstrated a positive correlation of elevated platelet distribution width (PDW) with a heightened likelihood of severe HT (19). As a new hematological indicator, PDW to platelet count ratio (PPR) can more comprehensively reflect platelet function, and its prognostic value has been confirmed in the prediction of other diseases (20, 21).

The objective of this study is to assess the predictive capabilities of various models utilizing different algorithms, develop a ML model that incorporates the PPR index for predicting the risk of HT after thrombolysis, and compare the performance of models to establish an effective assessment tool.

## 2 Methods

### 2.1 Study design and object

A single-center, observational, and retrospective study was conducted, and all subjects were collected from the First Affiliated Hospital of Kunming Medical University. The Ethics Committee of the hospital [No. 2022-L-157] provided approval for this study. Due to the retrospective nature of this study, which entailed anonymous and non-invasive data collection, the requirement for obtaining informed consent was waived. All procedures were performed in compliance with the principles outlined in the Declaration of Helsinki.

This study enrolled AIS patients who received IVT from July 2019 to April 2024. Subsequently, the patients were grouped into two groups, namely HT and non-HT, based on CT or MRI (magnetic resonance imaging) findings. A predictive model was constructed to assess the risk of HT following AIS.

**Figure 1:**
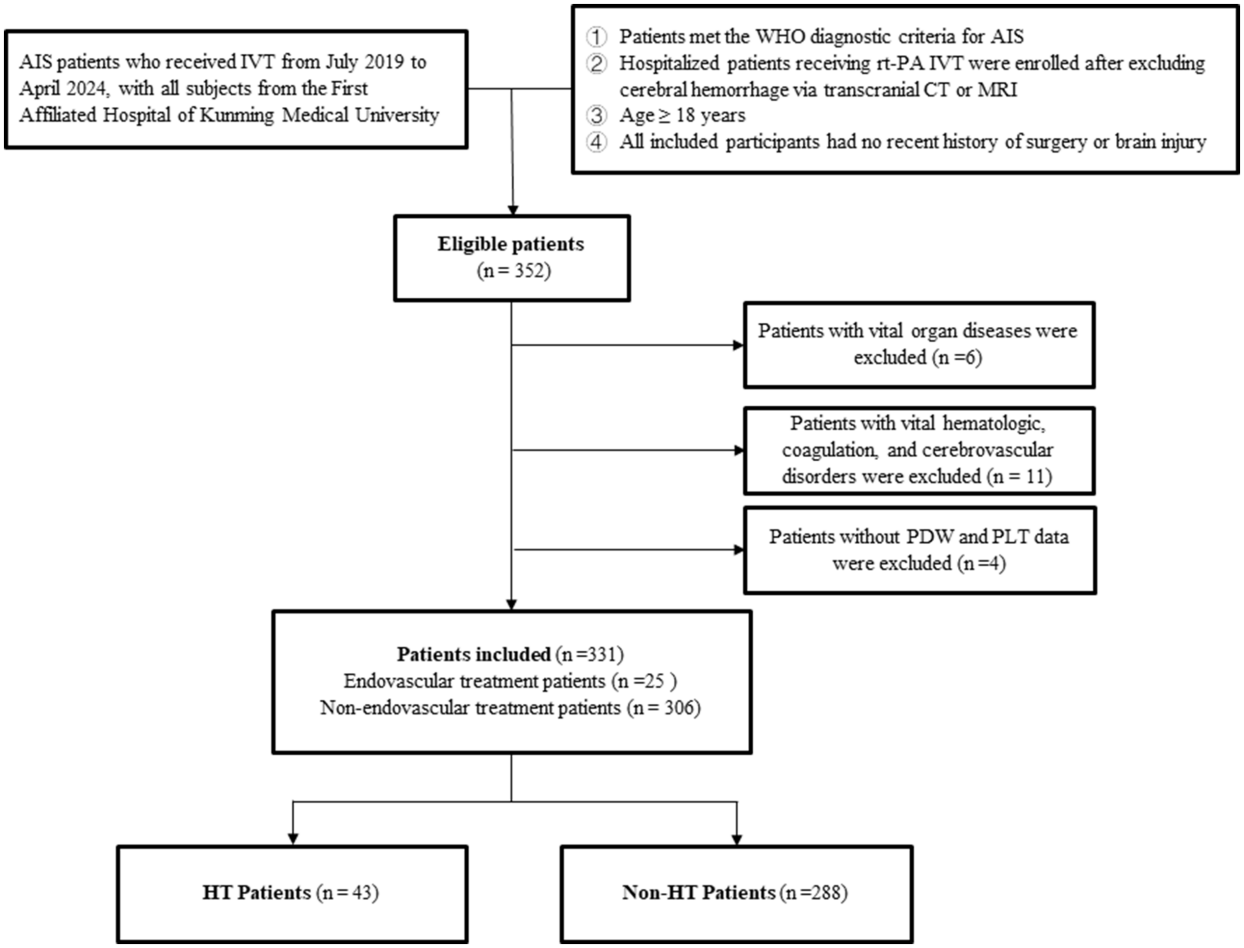
Patient selection process. AIS, Acute Ischemic Stroke; IVT, Intravenous Thrombolytic Therapy; PDW, Platelet Distribution Width; PLT, Platelet Count; HT, Hemorrhagic Transformation.

The inclusion criteria were as follows: (1) Patients who met the World Health Organization (WHO) diagnostic criteria for AIS; (2) Hospitalized patients who received rt-PA IVT after excluding cerebral hemorrhage through transcranial CT examination or magnetic resonance imaging; (3) Patients aged ≥ 18 years; (4) Participants without a recent history of surgical treatment or brain injury. The exclusion criteria were as follows: (1) Patients with concurrent vital organ diseases, such as liver and kidney impairment; (2) Patients complicated with blood system diseases, coagulation dysfunction, connective tissue diseases, cerebral aneurysms, and cerebrovascular malformations; (3) Patients lacked of PDW and PLT at admission (Figure 1).

### 2.2 Data collection and processing

The demographic data and clinical characteristics of the study participants (age, gender, diabetes, hypertension, atrial fibrillation, body mass index (BMI), smoking history, drinking history, malignant tumor, previous use of lipid-lowering drugs, previous use of antiplatelet drugs, previous use of anticoagulants, intravascular treatment after onset of disease, blood pressure, baseline National Institutes of Health Stroke Scale (NIHSS) score, time from onset to thrombolysis), along with their initial laboratory test results, which were first obtained before IVT initiation in AIS patients upon admission [blood routine, coagulation function, fibrinolysis, liver function, kidney function, electrolytes, blood lipids, glucose, myoglobin, brain natriuretic peptide (BNP)], were retrieved from the laboratory information system of the First Affiliated Hospital of Kunming Medical University. The PPR was calculated as (PDW/PLT). To predict missing values in continuous variables, a multiple imputation technique was utilized when the proportion of missing values was less than 20%. Categorical variables with more than 20% missing values were excluded. To mitigate multicollinearity, variables exhibiting a variance inflation factor (VIF) exceeding 5 were eliminated from the model.

### 2.3 LASSO regression for feature selection

LASSO regression was utilized to identify and select features significantly associated with HT, leveraging its ability to perform both variable selection and regularization. The primary strength of LASSO lies in its L1 regularization, which shrinks some regression coefficients to zero, thus effectively excluding irrelevant predictors. This automatic feature selection is conducive to focus the model with the most relevant variables related to HT, thus enhancing its interpretability. The optimal regularization parameter (*λ*) for LASSO was determined through cross-validation, a technique that helped to select the λ value and minimized model error by testing the model on different subsets of the data. By doing so, overfitting was mitigated, ensuring that the model generalized well to new data while still retaining the most meaningful predictors. Through this process of variable selection and regularization, LASSO improved both the accuracy and interpretability of the model, making it more effective for identifying significant predictors of HT.

### 2.4 Model construction

**Figure 2:**
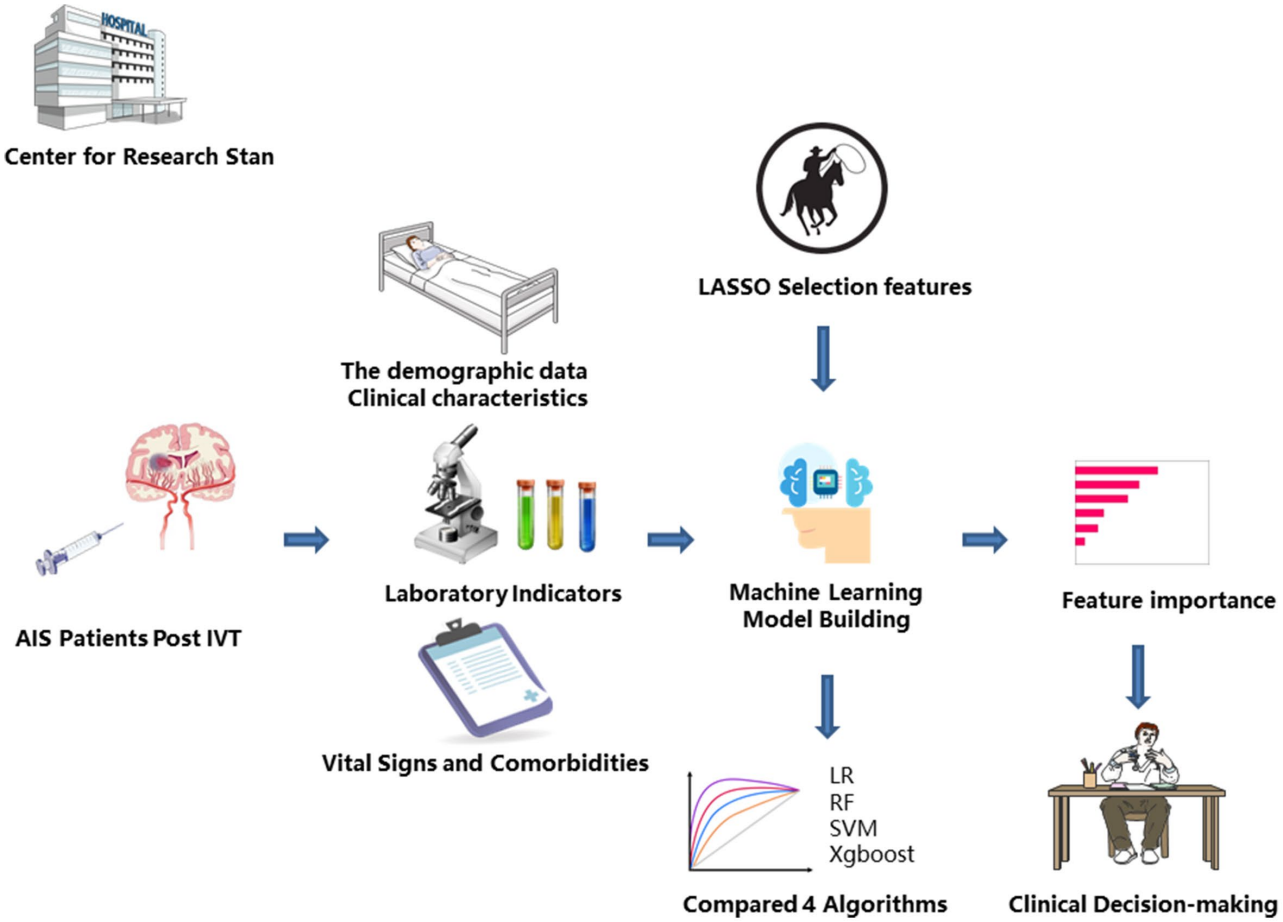
Machine learning flowchart for constructing predictive models. AIS, Acute Ischemic Stroke; IVT, Intravenous Thrombolytic Therapy; LR, Logistic Regression; RF, Random Forest; SVM, Support Vector Machine; xgboost, Extreme Gradient Boosting.

This retrospective study employed four widely used ML algorithms—Logistic Regression (LR), Random Forest (RF), Support Vector Machine (SVM), and xgboost—to predict the onset of HT following thrombolysis, as illustrated in Figure 2. The process began by selecting a set of significant features that were most strongly associated with the occurrence of HT. These features were then used as the input variables for training each of the four ML models. To ensure optimal model performance, hyperparameter tuning was conducted for each algorithm using a grid search approach. This method systematically explored different combinations of hyperparameters within a predefined parameter space specific to each algorithm. The models were then fine-tuned based on performance metrics obtained from an extensive search, ensuring that they were optimized to achieve their highest performance potential. For the four machine learning models (LR, RF, SVM, and xgboos), the following hyperparameters were fine-tuned: LR: The regularization strength (L1 or L2 regularization) was adjusted. RF: Hyperparameters such as the number of trees, maximum depth, and minimum samples required to split a node were optimized. SVM: The penalty parameter C and the type of kernel function were tuned.xgboos: Key hyperparameters like learning rate, number of trees, and maximum depth were adjusted. These hyperparameters were optimized through techniques like cross-validation, grid search, and random search, to find the optimal combination that maximized the model’s generalization ability on the validation set.

### 2.5 Training model

To train our models and mitigate the risk of overfitting, we implemented 5-fold cross-validation. This method involved dividing the dataset into 5 separate folds. This method involved dividing the dataset into 5 separate folds, maximizing the number of folds while ensuring that each fold contained a sufficient number of HT patient samples (8–9 positive samples per fold). In each round, the model was trained on 4 of the folds and validated on the remaining fold. The process was repeated 5 times, with each fold acting as the validation set once. The final model performance was calculated by taking the average of the metrics obtained from each iteration. By using this technique, the dataset was effectively split into 5 parts, and the model was trained and validated on different combinations of these parts. This helped to minimize potential bias in the performance assessment. As a result, this strategy provided a more robust and generalizable evaluation, producing performance metrics that were less reliant on any single partition of the data.

### 2.6 Model evaluation

To evaluate and ensure the generalizability of each model, the performance was assessed by calculating the mean [standard deviation (SD)] of key metrics across the 5-fold cross-validation, including the Area Under the Curve (AUC) of the Receiver Operating Characteristic (ROC), accuracy, sensitivity, specificity, and balanced F-score. These metrics provided a comprehensive evaluation of each model’s predictive capabilities. To explain the impact of predictors in a model, feature importance analysis was used.

### 2.7 Nomogram for HT prediction after IVT

The best-performing model, selected based on the highest AUC and overall metric scores, was used to identify the key features most strongly associated with HT. These selected features, along with the model’s predictions, were then incorporated into the development of a nomogram. The nomogram allows for the calculation of the probability of HT occurrence using multiple clinical variables. For each predictive variable, a horizontal line was drawn, with a scale beneath it indicating the possible values of that variable. Based on the actual observed value of each variable, the corresponding score was located on the scale. The scores for all variables were then summed to obtain a total score. This total score was finally mapped to a probability curve on the nomogram, allowing for the conversion of the total score into the predicted probability of HT occurrence in AIS patients who received IVT. The nomogram provided a visual, intuitive tool that allowed clinicians to estimate the probability of HT in individual patients following thrombolysis, facilitating decision-making and personalized care.

### 2.8 Statistical analysis

Continuous variables were represented as mean (SD) or median (upper and lower quartiles), and compared through student *t*-tests or non-parametric tests according to specific circumstances. Categorical variables were represented as frequency and percentage (%), and Pearson chi square test or Fisher’s exact test was adopted for comparison between groups. *p* < 0.05 was set to indicate a statistically significant difference. The statistical analysis of this study was performed using R software (version 4.3.2).

## 3 Results

### 3.1 Characteristics of patients

In this study, 331 AIS patients after IVT were included, of whom 43 (13.0%) developed HT. The patients had a median age of 68 [58, 77] years, and there were 205 (61.9%) males and 126 (38.1%) females. Differences were observed regarding the following variables between the HT group and the non-HT group: age, baseline NIHSS score, hemoglobin (Hb), BNP, D-dimer (D-D) (*p* < 0.05). The details are presented in Table 1.

**Table 1:** Clinical baseline characteristics.

Characteristic	Level	Overall	Non-HT	HT	*p*
Characteristics		331	288	43	
Age [median (IQR)]		68.00 [58.00, 77.00]	68.00 [57.00, 77.00]	72.00 [65.50, 82.00]	0.005
Gender (%)	Female	126 (38.1)	110 (38.2)	16 (37.2)	1
	male	205 (61.9)	178 (61.8)	27 (62.8)	
BMI [median (IQR)]		23.66 [22.04, 26.08]	23.66 [22.04, 26.03]	23.88 [20.39, 26.22]	0.736
Smoking (%)	None	228 (68.9)	195 (67.7)	33 (76.7)	0.309
	Yes	103 (31.1)	93 (32.3)	10 (23.3)	
Drinking (%)	None	275 (83.1)	238 (82.6)	37 (86.0)	0.735
	Yes	56 (16.9)	50 (17.4)	6 (14.0)	
Hypertension (%)	None	138 (41.7)	123 (42.7)	15 (34.9)	0.421
	Yes	193 (58.3)	165 (57.3)	28 (65.1)	
Diabetes (%)	None	251 (75.8)	224 (77.8)	27 (62.8)	0.051
	Yes	80 (24.2)	64 (22.2)	16 (37.2)	
CAD (%)	None	297 (89.7)	261 (90.6)	36 (83.7)	0.262
	Yes	34 (10.3)	27 (9.4)	7 (16.3)	
Stroke (%)	None	277 (83.7)	244 (84.7)	33 (76.7)	0.272
	Yes	54 (16.3)	44 (15.3)	10 (23.3)	
Arrhythmia (%)	None	309 (93.4)	269 (93.4)	40 (93.0)	1
	Yes	22 (6.6)	19 (6.6)	3 (7.0)	
Malignancy (%)	None	321 (97.0)	281 (97.6)	40 (93.0)	0.251
	Yes	10 (3.0)	7 (2.4)	3 (7.0)	
Antiplatelets (%)	None	280 (84.6)	246 (85.4)	34 (79.1)	0.396
	Yes	51 (15.4)	42 (14.6)	9 (20.9)	
Anticoagulants (%)	None	315 (95.2)	275 (95.5)	40 (93.0)	0.748
	Yes	16 (4.8)	13 (4.5)	3 (7.0)	
LLAs (%)	None	288 (87.0)	252 (87.5)	36 (83.7)	0.657
	Yes	43 (13.0)	36 (12.5)	7 (16.3)	
EVT (%)	None	306 (92.4)	268 (93.1)	38 (88.4)	0.438
	Yes	25 (7.6)	20 (6.9)	5 (11.6)	
OTT [median (IQR)] (h)		3.00 [2.10, 3.50]	3.00 [2.10, 3.60]	2.80 [1.85, 3.25]	0.124
NIHSS [median (IQR)]		5.00 [3.00, 10.00]	5.00 [3.00, 9.00]	14.00 [8.50, 18.00]	<0.001
SBP [mean (SD)] (mmHg)		143.51 (22.02)	143.07 (21.73)	146.42 (23.89)	0.353
DBP [median (IQR)] (mmHg)		84.00 [75.00, 93.00]	82.50 [75.00, 92.00]	88.00 [74.50, 95.50]	0.186
WBC [median (IQR)] (109/L)		7.52 [6.08, 9.22]	7.52 [6.04, 9.26]	7.62 [6.16, 9.16]	0.921
ANC [median (IQR)] (109/L)		4.97 [3.76, 6.84]	4.99 [3.76, 6.83]	4.77 [3.78, 6.84]	0.84
LYM [median (IQR)] (109/L)		1.59 [1.13, 2.13]	1.58 [1.13, 2.14]	1.67 [1.19, 2.09]	0.786
RBC [median (IQR)] (1,012/L)		4.85 [4.51, 5.24]	4.86 [4.55, 5.25]	4.69 [4.38, 5.15]	0.152
Hb [median (IQR)] (g/L)		149.00 [137.50, 161.00]	150.00 [139.00, 161.00]	141.00 [130.50, 156.50]	0.027
RDW-CV [median (IQR)] (%)		13.00 [12.80, 14.00]	13.00 [12.78, 14.00]	13.10 [12.90, 14.00]	0.267
CK-MB [median (IQR)] (ng/mL)		35.26 [22.18, 56.89]	34.98 [20.94, 56.72]	38.30 [28.24, 72.89]	0.16
Glucose [median (IQR)] (mmol/L)		7.13 [6.10, 8.95]	7.10 [6.10, 8.89]	7.30 [6.00, 10.07]	0.618
UA [mean (SD)] (μmol/L)		353.50 [275.10, 431.30]	354.05 [282.62, 433.55]	323.60 [238.90, 417.75]	0.188
Creatinine [median (IQR)] (μmol/L)		90.54 (54.95)	90.92 (57.99)	87.98 (27.05)	0.744
K + [median (IQR)] (mmol/L)		3.71 [3.46, 4.01]	3.70 [3.44, 4.01]	3.74 [3.50, 4.03]	0.649
Na + [median (IQR)] (mmol/L)		139.50 [137.47, 141.50]	139.52 [137.64, 141.49]	139.16 [137.00, 141.38]	0.482
Cl + [median (IQR)] (mmol/L)		103.92 [101.47, 106.55]	103.94 [101.70, 106.64]	103.72 [100.28, 106.16]	0.228
Ca2 + [median (IQR)] (mmol/L)		2.23 [2.17, 2.32]	2.24 [2.18, 2.33]	2.21 [2.16, 2.28]	0.056
Dbil [median (IQR)] (μmol/L)		4.00 [2.90, 5.80]	3.95 [2.80, 5.70]	4.60 [3.35, 6.85]	0.174
Ibil [median (IQR)] (μmol/L)		7.60 [5.20, 10.15]	7.50 [5.27, 10.12]	7.80 [5.05, 10.05]	0.686
ALB [median (IQR)] (g/L)		40.09 [37.45, 43.05]	40.10 [37.50, 43.02]	39.90 [36.35, 43.10]	0.731
BNP [median (IQR)] (pg/mL)		37.41 [10.84, 162.75]	31.24 [10.00, 155.10]	125.90 [51.66, 311.12]	<0.001
PTR [median (IQR)]		1.00 [0.97, 1.06]	1.00 [0.96, 1.06]	1.01 [0.97, 1.08]	0.653
PT [median (IQR)] (s)		13.10 [12.50, 13.80]	13.10 [12.50, 13.80]	13.20 [12.65, 13.70]	0.727
TT [median (IQR)] (s)		18.50 [17.60, 19.65]	18.60 [17.60, 19.70]	18.30 [17.60, 19.20]	0.462
INR [median (IQR)]		1.01 [0.96, 1.08]	1.00 [0.95, 1.08]	1.01 [0.96, 1.09]	0.687
FIB [median (IQR)] (g/L)		2.95 [2.58, 3.52]	2.95 [2.56, 3.50]	2.92 [2.68, 3.60]	0.626
D-D [median (IQR)] (mg/mL)		0.40 [0.25, 0.91]	0.36 [0.23, 0.85]	0.65 [0.32, 1.57]	0.003
PPR [median (IQR)]		0.07 [0.05, 0.09]	0.07 [0.05, 0.09]	0.07 [0.05, 0.09]	0.344

*p*, *p*-value from the statistical test comparing the HT and non-HT groups. BMI, Body Mass Index; CAD, Coronary Artery Disease; LLAs, Lower Limb Arterial Stenosis; EVT, Endovascular Therapy; OTT, Onset to Treatment Time; SBP, Systolic Blood Pressure; DBP, Diastolic Blood Pressure; WBC, White Blood Cell; ANC, Absolute Neutrophil Count; LYM, Lymphocytes; RBC, Red Blood Cell; Hb, Hemoglobin; RDW-CV, Red Cell Distribution Width-Coefficient of Variation; CK-MB, Creatine Kinase-MB; UA, Uric Acid; Dbil, Direct Bilirubin; Ibil, Indirect Bilirubin; ALB, Albumin; BNP, Brain Natriuretic Peptide; PTR, Prothrombin Ratio; PT, Prothrombin Time; TT, Thrombin Time; INR, International Normalized Ratio; FIB, Fibrinogen; D-D, D-dimer; PPR, Platelet Distribution Width to Platelet Count Ratio.

### 3.2 Selection of predictive variables

**Figure 3:**
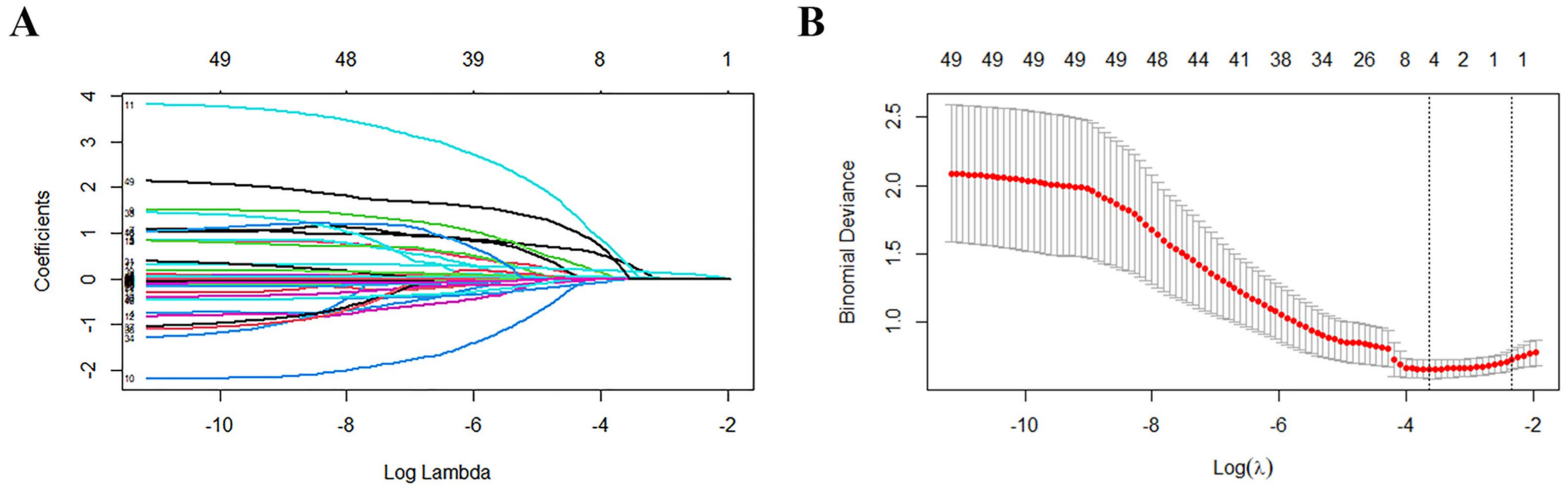
Results of variable screening using the LASSO regression. **(A)** The 5-fold cross-validation was performed, and the coefficients of all predictors gradually returned to zero. *λ* = 0.02651381. **(B)** There were 6 predictors of non-zero coefficients at the far right dotted line.

The function selection was carried out using the Least Absolute Shrinkage and Selection Operator (LASSO) method, where the penalty for *β* coefficient was determined by the tuning parameter *λ* (*λ* = 0.02651381). In this study, 37 variables were included, and 37 lines of different colors were obtained, each representing the change trajectory of a specific independent variable’s coefficient. As the value of *λ* increased, the coefficients gradually decreased, reflecting the regularization effect of the LASSO method (Figure 3A). The dashed line on the left represented λ value, and the value minimized the bias and corresponded to the optimal model fit. Regarding this value, the model selected 6 variables, indicating that these variables provided the most reliable and predictive relationship with the outcome. Consequently, six feature variables with non-zero coefficients were chosen, including age, diabetes, malignancy, onset to treatment time (OTT), baseline NIHSS score, and PPR (Figure 3B).

### 3.3 Model performance

**Figure 4:**
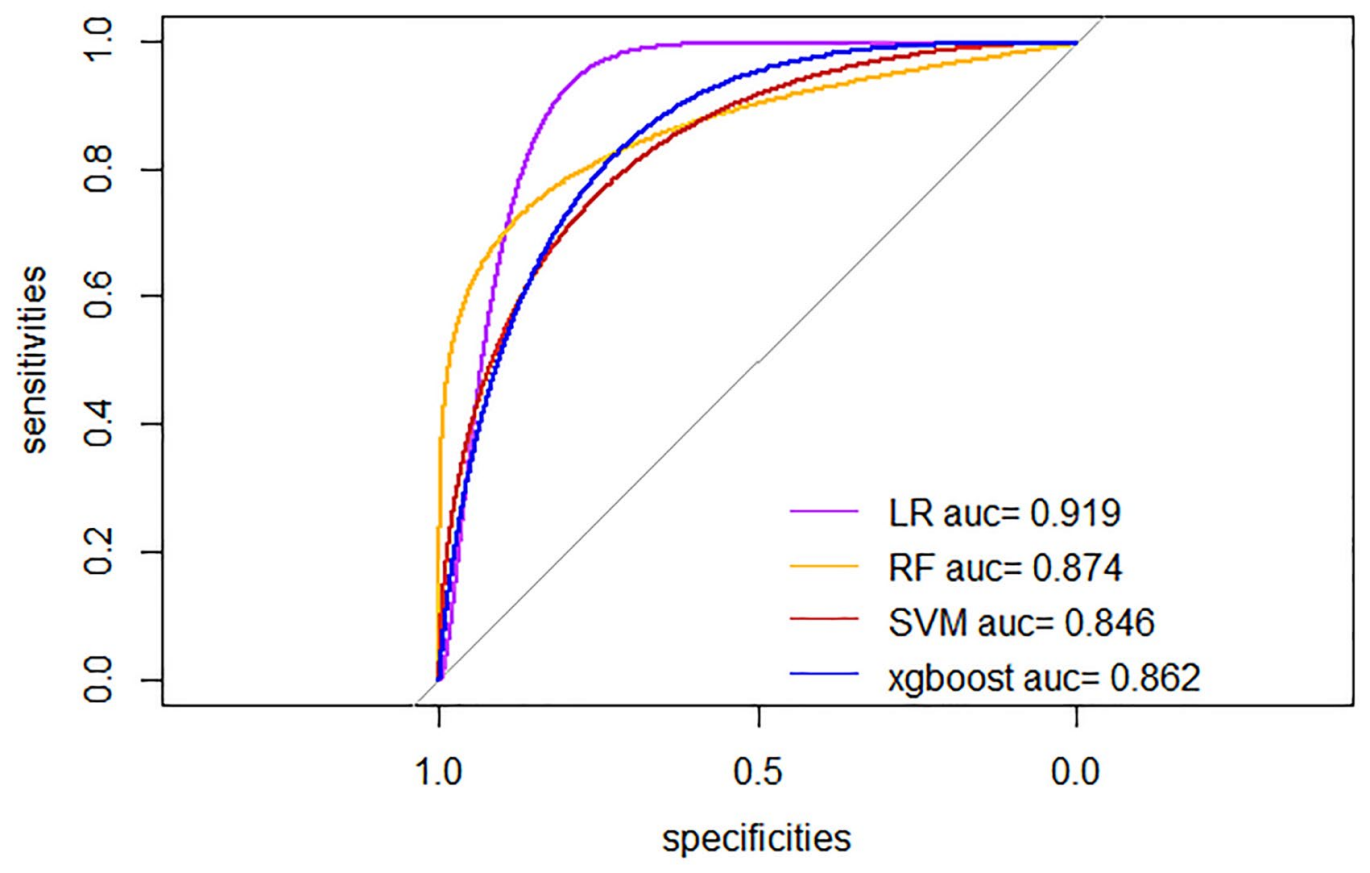
Optimal ROC curves for four ML models. LR, Logistic Regression; RF, Random Forest; SVM, Support Vector Machine; xgboost, Extreme Gradient Boosting.

Four ML algorithms, namely Logistic, Random Forest, SVM, and Xgboost, were selected to construct models. The optimized ML model underwent 5-fold cross-validation, and the mean value obtained from each algorithm was utilized as the prediction result for that algorithm. The mean accuracy values of LR, RF, SVM, and XGBoost models were 0.825, 0.743, 0.773, and 0.813, respectively; the mean AUC values were 0.851, 0.763, 0.711, and 0.718, respectively; the mean sensitivity values were 0.830, 0.821, 0.731, and 0.636, respectively; the mean specificity values were 0.832, 0.725, 0.776, and 0.841, respectively. The details are represented in Table 2. The optimal ROC curves for different models are shown in Figure 4. It could be found that the optimal AUC values for all four models were above 0.8 (LR > Xgboost>SVM > RF), indicating good fitting effect.

**Table 2:** Mean and standard deviation of 5-fold cross-validation for four ML Models.

Machine learning	AUC (mean ± SD)	Precision (mean ± SD)	Sensitivity (mean ± SD)	Specificity (mean ± SD)	Accuracy (mean ± SD)	F1 value (mean ± SD)
LR	0.851 ± 0.066	0.825 ± 0.081	0.830 ± 0.195	0.832 ± 0.122	0.477 ± 0.240	0.550 ± 0.136
RF	0.763 ± 0.084	0.743 ± 0.138	0.821 ± 0.142	0.725 ± 0.174	0.356 ± 0.127	0.481 ± 0.119
SVM	0.711 ± 0.115	0.773 ± 0.140	0.731 ± 0.143	0.776 ± 0.168	0.373 ± 0.11	0.479 ± 0.112
XGBoost	0.718 ± 0.104	0.813 ± 0.096	0.636 ± 0.234	0.841 ± 0.138	0.461 ± 0.175	0.478 ± 0.075

LR, Logistic Regression; RF, Random Forest; SVM, Support Vector Machine; XGBoost, Extreme Gradient Boosting.

**Figure 5:**
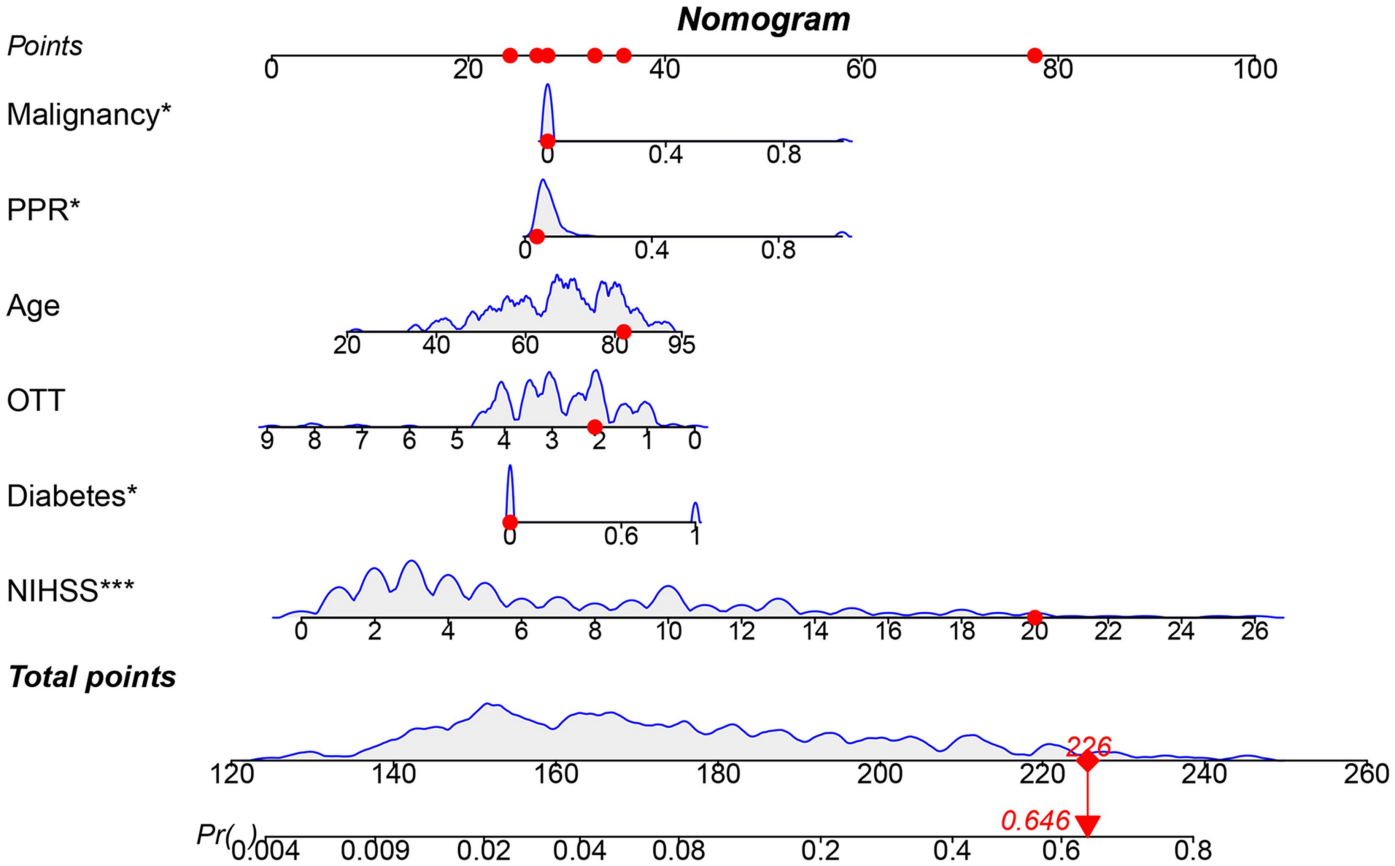
Nomogram model constructed based on LR. OTT, Onset to Treatment Time; PPR, Platelet Distribution Width to Platelet Count Ratio; Blue curve: Represents the relationship between one modeling variable and the occurrence of HT in AIS patients who received IVT; Gray shaded area: Represents the range of fluctuations in the occurrence of HT in AIS patients who received IVT as the input variables change.

**Figure 6:**
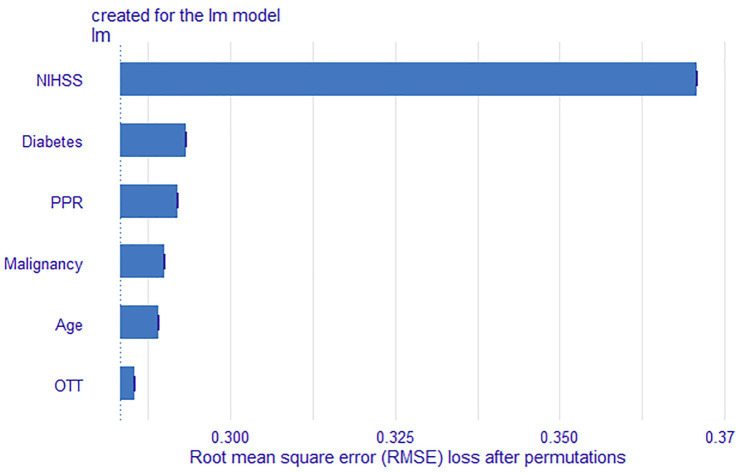
Feature importance results of the LR model. OTT, Onset to Treatment Time; PPR, Platelet Distribution Width to Platelet Count Ratio.

The results in Table 2 and Figure 5 indicated that after comprehensive evaluation of the four models, the LR model exhibited the best performance in terms of mean value of AUC, accuracy, sensitivity, precision, and F1. Therefore, it could be considered that the LR model had the best performance among these four models. According to the Nomogram constructed from the LR model and the statistical analysis of the LR mode, for AIS patients undergoing IVT, a total score of 226 corresponded to an estimated probability of 0.64 for HT (Figure 6).

### 3.4 Feature importance

The LR model demonstrated the best overall performance. We further ranked these features based on their contribution to the model’s predictive capability. The feature importance of the LR model are summarized in Figure 5. In addition, based on their contribution to the model, feature variables were ranked in descending order as baseline NIHSS score, diabetes, PPR, malignancy, age, and OTT.

## 4 Discussion

In this study, the occurrence of HT in AIS patients following IVT was found to be 13.0%, which was similar to the results in previous studies (22). The ML model based on patient PPR exhibited favorable performance in predicting HT, with optimal AUC values exceeding 0.8. Particularly, the LR model performed well in this study, with an optimal AUC value exceeding 0.9. In addition, the mean values of the model in accuracy, sensitivity, specificity, were all over 0.8. Overall, the LR model exhibited well in performance evaluation and model calibration, providing strong support in clinical decision-making. The reason LR may perform optimally compared to other models is that LR is a relatively simple linear model, particularly suitable for situations with small datasets and clear linear relationships between features. While other complex models like XGBoost have advantages in handling non-linear relationships, LR may demonstrate better predictive performance when dealing with linear data, less noise, or a lower risk of overfitting (23). Moreover, the ML-based predictive model developed in this study demonstrated superior risk prediction capabilities compared to previous MLR models (6). Compared with ML models developed by other researchers, the model developed by Wang et al. (24) was slightly inferior in terms of optimal performance (AUC = 0.82), and its inclusion of missing values in variables exceeded 30%, which might cause bias in HT prediction. The study by Li et al. (25) showed that the Xgboost model exhibited the highest performance in terms of AUC (AUC > 0.95). However, the CO2-CP included in this model was not a conventional testing index for AIS admission, which might also affect the promotion and application of the model in primary hospitals (25). The modeling variables in this study included age, diabetes, malignant tumor, OTT, baseline NIHSS score, and PPR. These were convenient for popularization. In summary, the HT risk predictive model developed in this study performed well in multiple performance indicators and had high clinical application potential compared to other ML models.

The role of platelets in ischemia–reperfusion injury has gained increasing attention in the pathophysiological process of AIS. The activation of platelets and activated platelets can exacerbate post-stroke ischemia–reperfusion injury, and the disruption of the blood–brain barrier by reperfusion injury is one of the important causes for HT (26–28). Platelet aggregation and clot retraction play important roles in the bleeding process. Among them, platelet aggregation is a key step in the hemostasis process, while clot retraction helps stabilize thrombosis and reduce the risk of hemorrhage (29). Alteplase can inhibit platelet aggregation and clot retraction by inhibiting ADP, collagen, and adrenaline, thereby affecting platelet function (30). Platelets can also enhance fibrinolysis by participating in the plasminogen activation system, thereby increasing the risk of HT after IVT (31). In addition, platelets form platelet-fibrin aggregates around the thrombus, leading to living contracting of cerebral thrombosis, thereby affecting the severity and prognosis of AIS (32). Mean platelet volume (MPV) and PDW together provide comprehensive information on platelet production, activation, and functional status. Compared to MPV, PDW is a more sensitive marker of variation in platelet volume, providing more comprehensive platelet activation information and effectively indicating the severity of the disease (19, 33, 34). In addition, Chen et al. (35) discovered an obvious association between PDW and the severity of stroke. Unfortunately, PDW may be affected by platelet count (36). Some scholars believe that PDW should not be used alone as a direct indicator of thromboembolic diseases (37). Lin et al.’s (21) study shows that the AUCs for predicting 120-day mortality in severe burn patients using PDW, PLT, and PPR on the third day post-burn are 0.792, 0.782, and 0.816, respectively. Therefore, as a novel biological indicator, PPR, by reflecting both the distribution width and platelet count, can more comprehensively reflect platelet function and predict the risk of HT occurrence. In this study, the baseline PPR lacked statistical significance, but *p*-values can be influenced by sample size and may not accurately reflect the true relationship between variables and outcomes, potentially leading to bias in variable selection. This is especially true when p-values are used as the sole criterion for feature selection, which may result in the inclusion of variables with no practical significance. In contrast, regularization methods like LASSO regression help address this issue by penalizing coefficients and automatically selecting variables, effectively removing less important ones and improving the model’s generalizability (38). Given that PPR was included in the LASSO model and ranked third in feature importance, its role as an independent impact factor is justifiable, as it demonstrated a certain effect in predicting HT. Future studies should further explore the predictive ability of PPR in different patient populations and evaluate its application value in clinical practice.

In our study, the key factors influencing the prediction results include malignant tumors and diabetes. Malignant tumor patients have a certain impact on the prediction results, which may be due to the higher coagulation, platelet, and endothelial dysfunction markers, as well as more circulating tumor microemboli in stroke patients complicated with malignant tumors (39). However, patients with malignant tumors may not necessarily develop HT (40–42). Other variables still need to be explored for comprehensive evaluation. In addition, in our model, diabetes may have an important influence on the prediction results (Ranked 2rd in feature importance), which may be due to the combined effect of multiple mechanisms such as endothelial dysfunction, changes in coagulation and fibrinolytic systems, abnormal platelet function, and direct tissue damage caused by hyperglycemia in diabetes patients (43). Previous studies have shown that the admission glucose performs better in predicting the adverse outcome of AIS patients than diabetes (44, 45). It should be noted that there are differences in physiological mechanism between chronic hyperglycemia and stress hyperglycemia (46). Therefore, it may not fully reflect the actual condition of patients to consider diabetes and admission glucose alone. Future studies should further explore biomarkers and clinical parameters that reflect comprehensive blood glucose levels, and construct more accurate predictive models. By incorporating age, OTT, and baseline NIHSS score into the model, the findings of previous studies have been effectively corroborated and validated (5, 47–49).

Imaging variables may encounter certain challenges in predicting HT risk after IVT. Although IVT can effectively dissolve thrombus and restore cerebral blood flow, it also increases the risk of intracranial hemorrhage (50). Therefore, when deciding to perform IVT on AIS patients, the risk of HT is one of the primary factors that should be considered by clinicians. IVT is suitable for AIS patients within 3 to 4.5 h after onset (51), which requires rapid and accurate evaluation by clinicians. Imaging plays a pivotal role in the rapid diagnosis and treatment of ischemic stroke. Head computed tomography (CT) scan can quickly and accurately determine cerebral hemorrhage; CT angiography (CTA) can locate ischemic blood vessels; CT perfusion (CTP) imaging can detect ischemic penumbra through multiple automated post-processing; MRI and diffusion-weighted imaging (DWI) can clarify the diagnosis of AIS and the extent of cerebral infarction (52). Due to the high risk of radiation exposure and contrast agent application of CT and CTA, as well as the longer duration, higher cost, and limited equipment accessibility of CTP and MRI, it may lead to different imaging protocols chosen by clinicians, resulting in different imaging variables. MRI and CTP may have moderate diagnostic performance in predicting HT in patients with AIS (53, 54), but current clinical evidence is insufficient to support these imaging parameters in predicting HT (55). Therefore, challenges still exist in incorporating imaging variables into predictive models to assess the risk of HT after thrombolysis. Moreover, multi-center studies have shown that early active treatment and dehydration therapy for asymptomatic HT patients can reduce the risk of hematoma enlargement and death (4). This study aims to develop a model based on ML combined with laboratory indicators that enables rapid and accurate prediction of HT following IVT. This will assist clinicians in making informed decisions regarding the administration of thrombolytic therapy and facilitate the early identification of asymptomatic HT patients after IVT, so as to prevent them from developing PH. By comprehensively analyzing various clinical and laboratory data, and combining with ML algorithms, the predictive model developed in this study has been able to efficiently and accurately evaluate the risk of HT (optimal AUC > 0.9). Future studies will focus on standardizing multiple imaging variables to further optimize the predictive ability of HT.

### 4.1 Limitations

This study used single-center data, lacked external validation, and adopted a retrospective study design, which could potentially limit the generalizability and accuracy of the research findings. Additionally, while HT was assessed as a whole, it was not further divided into its subtypes—HI and PH. PH was generally associated with more severe outcomes and poorer prognosis compared to HI, making it a critical factor for risk stratification and prediction in ischemic stroke patients. Future studies should aim to distinguish between HI and PH to better predict and manage the more severe forms of hemorrhagic transformation. Incorporating multi-center data and adopting prospective designs would also improve the generalizability and accuracy of predictive models.

## 5 Conclusion

It can be concluded in our research that the independent predictors of HT are age, diabetes, malignancy, OTT, baseline NIHSS score, and PPR. Among the models constructed by four ML algorithms, we have chosen the HT model with the best performance constructed by the LR algorithm. This model offers precise predictions of HT after IVT, providing valuable support to clinicians in promptly and accurately assessing the risk of thrombolytic hemorrhage and identifying asymptomatic HT patients after IVT.

## Data Availability

The original contributions presented in the study are included in the article/supplementary material, further inquiries can be directed to the corresponding author.

## Glossary

HT: Hemorrhagic transformation
AIS: acute ischemic stroke
IVT: intravenous thrombolytic therapy
ML: machine learning
PPR: platelet count ratio
AUC: area under curve
ROC: receiver operating characteristic
OTT: onset to treatment time
HI: hemorrhagic infarction
PH: parenchymal hemorrhage
PDW: platelet distribution width
WHO: World Health Organization
BMI: body mass index
NIHSS: National Institutes of Health Stroke Scale
BNP: brain natriuretic peptide
VIF: variance inflation factor
SD: standard deviation
MPV: Mean platelet volume
CT: computed tomography
CTA: CT angiography
CTP: CT perfusion
DWI: diffusion-weighted imaging
